# Haplotyping a Quantitative Trait with a High-Density Map in Experimental Crosses

**DOI:** 10.1371/journal.pone.0000732

**Published:** 2007-08-15

**Authors:** Wei Hou, John Stephen F. Yap, Song Wu, Tian Liu, James M. Cheverud, Rongling Wu

**Affiliations:** 1 Department of Epidemiology and Health Policy Research, University of Florida, Gainesville, Florida, United States of America; 2 Department of Statistics, University of Florida, Gainesville, Florida, United States of America; 3 Department of Anatomy and Neurobiology, Washington University Medical School, St. Louis, Missouri, United States of America; Vrije Universiteit Medical Centre, Netherlands

## Abstract

**Background:**

The ultimate goal of genetic mapping of quantitative trait loci (QTL) is the positional cloning of genes involved in any agriculturally or medically important phenotype. However, only a small portion (≤ 1%) of the QTL detected have been characterized at the molecular level, despite the report of hundreds of thousands of QTL for different traits and populations.

**Methods/Results:**

We develop a statistical model for detecting and characterizing the nucleotide structure and organization of haplotypes that underlie QTL responsible for a quantitative trait in an F_2_ pedigree. The discovery of such haplotypes by the new model will facilitate the molecular cloning of a QTL. Our model is founded on population genetic properties of genes that are segregating in a pedigree, constructed with the mixture-based maximum likelihood context and implemented with the EM algorithm. The closed forms have been derived to estimate the linkage and linkage disequilibria among different molecular markers, such as single nucleotide polymorphisms, and quantitative genetic effects of haplotypes constructed by non-alleles of these markers. Results from the analysis of a real example in mouse have validated the usefulness and utilization of the model proposed.

**Conclusion:**

The model is flexible to be extended to model a complex network of genetic regulation that includes the interactions between different haplotypes and between haplotypes and environments.

## Introduction

The basic principle for quantitative trait locus (QTL) mapping is the cosegregation of the alleles at a QTL with those at one or a set of known polymorphic markers genotyped on a genome in an experimental cross [1], [2]. If a QTL is cosegregating with molecular markers, the genetic effects of the QTL on a quantitative trait and its genomic position can be estimated from the marker genotypes and phenotypic values of the trait. This estimation process particularly assumes the QTL to be located within an interval constructed by a pair of flanking markers in which a test statistics calculated under the reduced (there is no QTL) and full model (there is a QTL) is used to test the existence of the QTL and estimate its position. This so-called interval mapping approach and its extensions [3]–[5] is robust and powerful for the detection of major QTL and presents the most efficient way to utilize marker information when marker maps are sparse [6]. However, interval mapping is limited by its incapacity to infer any information about the sequence structure and organization of the QTL. Partly for this reason, only a few QTL mapped from markers have been successfully cloned [7]–[9], despite a considerable number of QTL reported in the literature.

Interval QTL mapping also has an unsolved statistical difficulty when it is used with a high-density linkage map. With more markers genotyped, a genetic map for QTL identification has tended to be infinitely dense. For such an infinitely dense map in which markers are located everywhere over the genome, test statistics at nearby intervals are not independent any more. Thus, the critical threshold used to acclaim the existence of a QTL by interval mapping will be difficult to analytically determine. Although an empirical alternative based on permutation tests has been proposed for threshold determination [10], extensive computing may affect the use efficiency of interval mapping.

Despite its unsuitability for interval mapping of QTL, an infinitely dense map provides an important tool for characterizing genetic variants that contribute to quantitative variation via the analysis of haplotypes composed of non-alleles at a set of highly linked markers. Recent genetic studies suggest that a gene may determine a complex trait, such as body weight or drug response, through its haplotype rather than genotype [11], [12]. The completion of the genome projects for several important organisms, Arabdopsis, chicken, human, mouse and poplar, has made massive amounts of DNA sequence data available. In particular, single nucleotide polymorphisms (SNPs), being the most common type of variant in the DNA sequence, provide a powerful means for genotyping the whole genome or any part of it. This facilitates the identification of specific SNP-constructed haplotypes which are responsible for quantitative traits. A set of SNPs that cause quantitative differences among individuals are called quantitative trait nucleotides (QTNs). Liu et al. [13] proposed a statistical model for estimating and testing haplotype effects at a QTN in a random sample drawn from a natural population. This model is based on the population genetic properties of gene segregation. Through the implementation of the EM algorithm, population genetic parameters of SNPs, such as haplotype frequencies, allele frequencies and linkage disequilibria, and quantitative genetic parameters, such as haplotype effects of a QTN, are estimated with closed forms.

The motivation of this work is to derive a statistical model for haplotype discovery responsible for quantitative variation in a mapping population derived an experimental cross. Unlike a natural population in which gene co-segregation analysis is based on linkage disequilibria [14], experimental crosses, such as the backcross or F_2_, have usually been analyzed in terms of the linkage between different markers and QTL. In this article, we will frame a general statistical model for estimating the linkage between different SNPs and testing haplotype effects within the context of linkage disequilibrium analysis in an F_2_ pedigree. We show that the new model can test for the dependence of SNPs when a multi-point analysis is performed. We have derived closed forms for the EM algorithm to estimate a variety of genetic parameters. A worked example is used to validate the usefulness and utilization of the model.

## Methods

### Haplotype and diplotype

**Figure 1:**
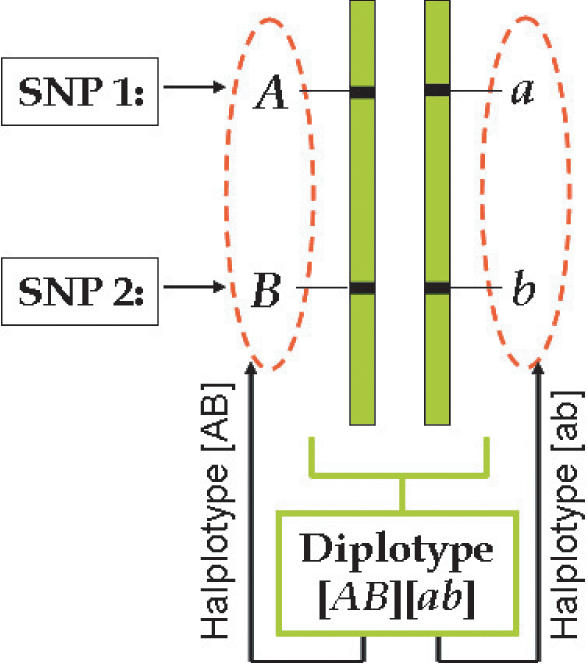
Haplotype configuration of a diplotype for two hypothesized SNPs.

A haplotype represents a linear arrangement of nucleotides (alleles) at different SNPs on a single chromosome, or part of a chromosome. The pair of haplotypes is called a diplotype. The observed phenotype of a diplotype is called a genotype. A diplotype is always constructed by two haplotypes, one from the maternal parent and the other from the paternal parent. Suppose there are two different SNPs on the same genomic region, one with two alleles *A* and *a* and the other with two alleles *B* and *b*, respectively. Allele *A* from SNP 1 and allele *B* from SNP 2 are located on the first homologous chromosome, whereas allele *a* from SNP 1 and allele *b* from SNP 2 located on the second homologous chromosome. Thus, [*AB*] is one haplotype and [*ab*] is a second haplotype, and both constitute a diplotype [*AB*][*ab*] (Fig. 1).

**Figure 2:**
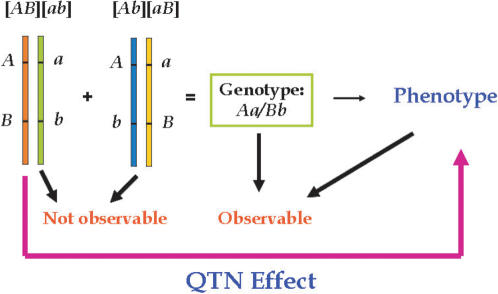
Diplotype configuration of a genotype for two hypothesized SNPs.

In a practical genetic analysis, we can only observe the genotype expressed as *Aa/Bb*. However, the double heterozygote may be one (and only one) of two possible diplotypes [*AB*][*ab*] and [*Ab*][*aB*]. But these two diplotypes cannot be directly observed and should be inferred from SNP genotype data (Fig. 2). In practice, it is important to estimate haplotype effects on a quantitative trait based on the diplotypes and therefore genotypes. For example, if an animal carries haplotype [*AB*], it will grow better than other animals that carries any other haplotypes, [*Ab*], [*aB*] and [*ab*]. For this reason, the same genotype *Aa/Bb* may perform differently, depending on what diplotype it carries. If this genotype is diplotype [*AB*][*ab*], then it will have a better growth. If the animal is diplotype [*Ab*][*aB*], its growth will be poorer. The statistical model being developed will be used to determine which diplotype is associated with better growth in experimental crosses.

### Linkage disequilibrium in the F_2_ intercross

**A general model:** Haplotype analysis in the backcross is straightforward because the diplotype is determined for all the backcross genotype. Simple analysis of variance can be used to detect haplotype effects on a quantitative trait. In the F_2_, this is not a case in which the double heterozygote is a mixture of two possible diplotypes.

Suppose many SNPs are genotyped each of which is segregating in a 1:2:1 Mendelian ratio in the F_2_ population. As seen in the human genome [15], these SNPs are divided into different haplotype blocks. For a given block, there are a particular number of representative SNPs or htSNPs that uniquely identify the common haplotypes in this block or QTN. Several algorithms have been developed to identify a minimal subset of htSNPs that can characterize the most common haplotypes [16]–[18]. Consider a QTN that contains *L* htSNPs among which there exist linkage disequilibria of different orders. The two alleles, 1 and 0, at each of these SNPs are symbolized by *r*_1_,…,*r_L_*, respectively. For a cross initiated with two inbred parents, the allele frequencies for each of these htSNPs should be 1/2. A haplotype frequency, denoted as 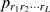, is decomposed into the following components:
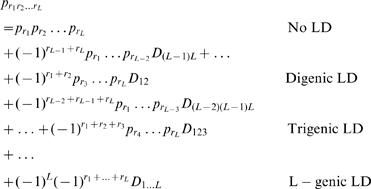
(1)where *D*'s are the linkage disequilibria of different orders among particular SNPs.

Totally, *L* SNPs form 2*^L^* haplotypes expressed as [*r*_1_…*r_L_*], 2*^L^* ^−1^(2*^L^*+1) diplotypes, i.e., a pair of maternally- (m) and paternally-derived haplotypes (p), expressed as [*r*_1_^m^…*r_L_* ^m^][*r*_1_^p^…*r_L_* ^p^] (*r*_1_^m^, *r*_1_^p^,…;*r_L_* ^m^,*r_L_* ^p^ = 1,0) and 3*^L^* genotypes expressed as *r*_1_ *r*′_1_/…/*r_L_r*′*_L_* (*r*_1_≥*r*′_1,_…,*r_L_*≥*r*′*_L_ = *1,0). Only genotypes can be observed. The number of diplotypes is smaller than the number of genotypes because the genotypes that are heterozygous at two or more SNPs contain multiple different diplotypes. Diplotype (and therefore genotype) frequencies can be expressed in terms of haplotype frequencies. We use 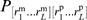 and 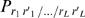 to denote the diplotype and genotype frequencies, respectively, and 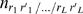 to denote genotype observation.

**A special case: Two-point linkage disequilibrium:** For two given SNPs (**S**_1_ and **S**_2_), there are four different haplotypes in a cross population. According to the definition given above, these four haplotypes are denoted as [11], [10], [01] and [00], whose frequencies in a cross population are, respectively, expressed as 
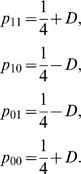
(2)Assume that the two SNPs are linked with a recombination fraction *r*. The haplotype frequencies can be expressed in terms of *r*, i.e., 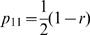, 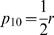, 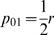 and 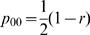. Combining equation (2), this establishes the relation between the linkage disequilibrium and recombination fraction as 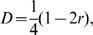
(3)or
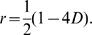
(4)


**A special case: Three-point linkage disequilibrium:** For three given SNPs (**S**_1,_ **S**_2, _and **S**_3_), there are eight different haplotypes, i.e., [111], [110], [101], [100], [011], [010], [001], and [000]. The haplotype frequencies in a cross population are, respectively, expressed as
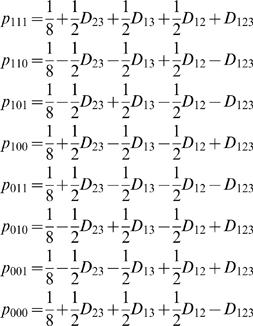
(5)where *D*_12_, *D*_23_ and *D*_13_ are the linkage disequilibria between SNP **S**_1_ and **S**_2_, between **S**_2_ and **S**_3_ and between **S**_1_ and **S**_2_, respectively, and *D*_123_ is the linkage disequilibrium among the three SNPs. The four disequilibrium coefficients can be estimated, by solving equation (5), as
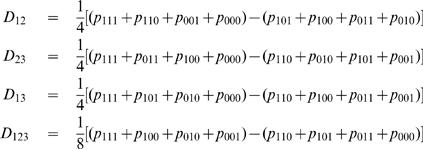
(6)The first three first-order linkage disequilibria can be used to describe the linkage between different SNPs and crossover interference, whereas the last second-order linkage disequilibrium is thought to be associated with chromatid interference.

### Haplotyping a trait with two SNPs

Our interest is to search for the haplotype diversity that can explain phenotypic variation in a complex trait. The association between haplotype diversity and phenotypic variation has been detected in several studies of drug responses [11], [12]. This allows us to assume that a particular haplotype is different from other haplotypes for a given trait. Here, our focus will be on modelling haplotype effects in experimental crosses. Although haplotypes (comprising diplotypes) can be directly observed in the backcross, this is not possible for the F_2_ because their heterozygous genotypes are not concordant with diplotypes or haplotypes. For the F_2_ population, the effects of different haplotypes on the phenotype need be postulated from observed zygotic genotypes. The inference of diplotypes for a particular genotype is statistically a missing data problem that can be formulated by a finite mixture model.

**Mixture model:** The statistical method for the genomewide scan of QTN is formulated on the basis of a finite mixture model. The mixture model assumes that each observation comes from one of an assumed set of distributions. The mixture model derived to detect haplotype effects on a quantitative trait based on SNP genotype data contains three major parts: (1) the mixture proportions of each distribution, denoted as the relative frequencies of different diplotypes for the same SNP genotype, (2) the mean for each diplotype in the density function, and (3) the residual variance common to all diplotypes.

For simplicity, we consider a QTN that is composed of only two SNPs each with two alleles designated as 1 and 0. These two SNPs segregating in the F_2_ population form four haplotypes whose frequencies are arrayed in vector Θp = (*p*_11_, *p*_10_, *p*_01_, *p*_00_). All the genotypes are consistent with diplotypes, except for the double heterozygote, 10/10, that contains two different diplotypes [11][00] with a frequency of 2 *p*_11_ *p*_00_ and [10][01] with a frequency of 2 *p*_10_ *p*_01_ (Table 1). The relative frequencies of different diplotypes for the double heterozygote are a function of haplotype frequencies.

**Table 1:** Diplotypes and their frequencies for each of nine genotypes at two SNPs within a QTN, haplotype composition frequencies for each genotype, and composite diplotypes for four possible risk haplotypes.

Genotype	Diplotype		Relative diplotype frequency	Risk haplotype			
	Configuration	Frequency		[11]	[10]	[01]	[00]
11/11	[11][11]	*p* ^2^_11_	1	*AA*	*A̅* *A̅*	*A̅* *A̅*	*A̅* *A̅*
11/10	[11][10]	2*p*_11_ *p*_10_	1	*AA̅*	*AA̅*	*A̅* *A̅*	*A̅* *A̅*
11/00	[10][10]	*p* ^2^_10_	1	*A̅* *A̅*	*AA*	*A̅* *A̅*	*A̅* *A̅*
10/11	[11][01]	2*p*_11_ *p*_01_	1	*AA̅*	*A̅* *A̅*	*AA̅*	*A̅* *A̅*
10/10	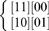	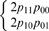	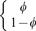	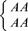	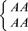	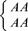	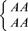
10/00	[10][00]	2*p*_10_ *p*_00_	1	*A̅* *A̅*	*AA̅*	*A̅* *A̅*	*AA̅*
00/11	[01][01]	*p* ^2^_01_	1	*A̅* *A̅*	*A̅* *A̅*	*AA*	*A̅* *A̅*
00/10	[01][00]	2*p*_01_ *p*_00_	1	*A̅* *A̅*	*A̅* *A̅*	*AA̅*	*AA̅*
00/00	[00][00]	*p* ^2^_00_	1	*A̅* *A̅*	*A̅* *A̅*	*A̅* *A̅*	*AA*

Two alleles for each of the two SNPs are denoted as 1 and 0, respectively. Genotypes at different SNPs are separated by a slash. Diplotypes are the combination of two bracketed maternally and paternally derived haplotypes. By assuming different haplotypes as a risk haplotype, composite diplotypes are accordingly defined and their genotypic values are given.

A total of *n* individuals in the F_2_ are classified into 9 genotypes for the two SNPs, each genotype with observation generally expressed as 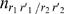 (*r*_1_≥*r*′_1_,*r*_2_≥*r*′_2_,*r*_3_≥*r*′_3_ = 1,0). The frequency of each genotype can be expressed in terms of haplotype frequencies (Table 1). Considering a quantitative trait controlled by diplotype (rather than genotype) diversity, the phenotypic value of the trait (*y_i_*) for individual *i* is expressed by a linear model, i.e.,
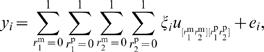
(7)where *ξ_i_* is the indicator variable defined as 1 if a diplotype considered is compatible with subject *i* and as 0 otherwise, 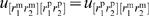 is the genotypic value for diplotype 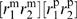, and *e_i_* is the residual error distributed as *N*(0,*σ* ^2^).

Assume that this QTN triggers an effect on the trait because at least one haplotype is different from the remaining seven. Without loss of generality, let [11] be such a distinct haplotype, called *risk haplotype*, designated as *A*. All the other non-risk haplotypes, [10], [01] and [00], are collectively expressed as *A̅*. The risk and non-risk haplotypes form three *composite diplotypes AA* (**2**), *AA̅* (**1**) and *A̅* *A̅* (**0**). Let *μ*_2_, *μ*_1 _and *μ*_0_ be the genotypic value of the three composite diplotypes, respectively (Table 1). The means for different composite diplotypes and residual variance are arrayed by a quantitative genetic parameter vector Θ*_q_* = (*μ*_2,_ *μ*_1,_ *μ*_0,_ *σ^2^)*.

**Likelihoods:** With the above notation, we construct two likelihoods, one for haplotype frequencies (Θ*_p_*) based on SNP data (**S**) and the other for quantitative genetic parameters (Θ*_q_*) based on haplotype frequencies (Θ*_p_*), phenotypic (*y*) and SNP data (**S**). They are, respectively, expressed as
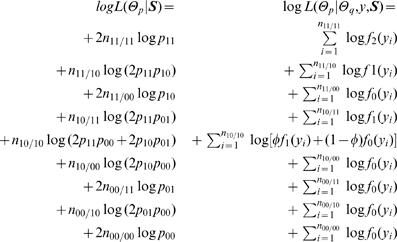
(8)where *f _j_* (*y_i_*) is a normal distribution density function of composite diplotype *j* (*j* = 2,1,0), i.e.,
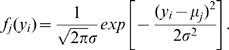
It can be seen from the above likelihood functions that, although most zygote genotypes contain a single component (diplotype), the double heterozygote is the mixture of two possible diplotypes weighted by *φ* and 1-*φ*, expressed as
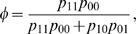
(9)which represents the relative frequency of diplotype [11][00] for the double heterozygote.

It should be noted that *L*(Θ*_p_*, Θ*_q_* | *y*, **S**) relies on the haplotype frequencies defined in *L*(Θ*_p_*|**S**) and, thus, the latter is thought to be nested within the former. The estimates of parameters that maximize *L*(Θ*_p_*|**S**) can also maximize the *L*(Θ*_p_*, Θ*_q_* | *y*, **S**).

**The EM algorithm:** A closed-form solution for the EM algorithm has been derived to estimate the unknown parameters that maximize the two likelihoods of (26) [13]. The estimates of haplotype frequencies are based on the log-likelihood function *L*(Θ*_p_*|**M**), whereas the estimates of diplotype genotypic means and residual variance are based on the log-likelihood function *L*(Θ*_p_*, Θ*_q_* | *y*, **M**). These two different types of parameters can be estimated using a two-stage hierarchical EM algorithm.

At a higher hierarchy of the EM algorithm, the E step is aimed to calculate the relative frequency (*φ*) of diplotype [11][00] in the double heterozygote is calculated by equation (9). The M step is aimed to estimate the haplotype frequencies based on the probabilities calculated in the previous iteration using
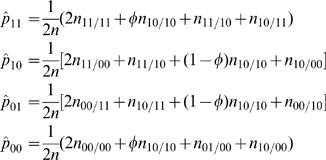
(10)


At a lower hierarchy of the EM algorithm, the E step is derived to calculate the posterior probability (Ω[_11_]_[00]*i*_) of individual *i* with the double heterozygous genotype to be diplotype [11][00] by
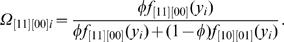
Note that for all the other genotypes, such posterior probabilities do not exist.

By assuming that [11] is a risk haplotype, the M step is derived to estimate the genotypic values (*μ_j_*) for each composite diplotype and the residual variance based on the calculated posterior probabilities by
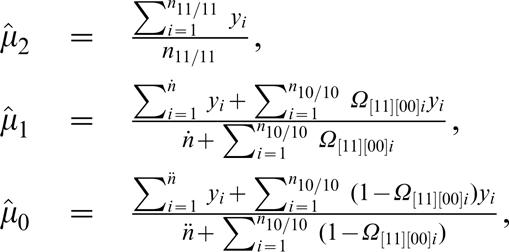
(12)

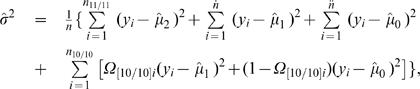
(13)where
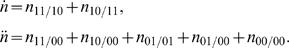
Iterations including the E and M steps are repeated at the higher hierarchy between equations (9) and (10) and at the lower hierarchy among equations (12) and (13) until the estimates of the parameters converge to stable values. The sampling errors of these parameters can be estimated by calculating Louis' [19] observed information matrix.

Haplotype frequencies can be expressed as a function of allelic frequencies and linkage disequilibrium. Based on equation (2), we solve the linkage disequilibrium between two SNPs by
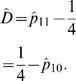
(14)With the genotypic means of composite diplotypes, we can estimate the overall mean (*μ*) and additive (*a*) and dominant genetic effects (*d*) due to the QTN detected, respectively, by
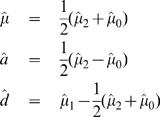



**Model selection:** The likelihood *L*(Θ*_p_*, Θ*_q_* | *y*, **S**) is formulated by assuming that haplotype [11] [11] is a risk haplotype. However, a real risk haplotype is unknown from raw data (*y*, **S**). An additional step for the choice of the most likely risk haplotype should be implemented. The simplest way to do so is to calculate the likelihood values by assuming that any one of the four haplotypes can be a risk haplotype (Table 1). Thus, we obtain four possible likelihood values under different risk haplotypes; that is, (1) 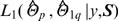 for [11], (2) 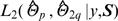 for [10], (3) 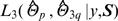 for [01], and (4) 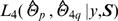 for [00]. Under each possible risk haplotype, we estimate the quantitative genetic parameters 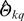 (*k* = 1,…,4). The largest likelihood value calculated is thought to correspond to the most likely risk haplotype.

In practice, it is also possible that there exist more than one risk haplotypes for a QTN. Relative to the bi-“allelic” QTN with one risk haplotype, such a QTN is called a multi-“allelic” QTN. If there are two risk haplotypes, we will have six composite diplotypes. Assuming that [11] (denoted by *A*_1_) and [10] (denoted by *A*_2_) are risk haplotypes and the remaining haplotypes [10] and [01] are non-risk haplotypes (denoted by *A*_3_), then six composite diplotypes, expressed as *A*_1_ *A*_1_, *A*_1_ *A*_2_, *A*_1_ *A*_3_, *A*_2_ *A*_2_, *A*_2_ *A*_3_ and *A*_3_ *A*_3_, can be specified according to the diplotype distribution as shown in Table 1. Totally, there are six such haplotype combinations for a two-SNP QTL, each combination corresponding to a likelihood value. Based on the calculated likelihoods, we can determine a most likely risk and non-risk haplotype combination. If there are three risk haplotypes, we will have 10 different composite diplotypes. The optimal risk and non-risk haplotype combination will be selected from three combinations based on the likelihoods.

The likelihood can be used as a criterion to select the optimal risk and non-risk haplotype combination when the number of risk haplotype is the same. However, when the number of risk haplotype is different, an AIC- or BIC-based model selection strategy [20] should be used because of different numbers of parameters being estimated in this case.

**Hypothesis tests:** We can test two major hypotheses in the following sequence: (1) the association between two SNPs by testing their linkage disequilibrium, and (2) the difference of a given haplotype from the remaining haplotypes by testing the significance of haplotype additive and dominant effects on the trait. The linkage disequilibrium between two given SNPs can be tested using two alternative hypotheses:
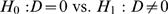
(15)The log-likelihood ratio test statistic for the significance of LD is calculated by comparing the likelihood values under the *H_1_* (full model) and *H_0_* (reduced model) using
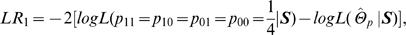
(16)The *LR_1_* is considered to asymptotically follow a χ^2^ distribution with one degree of freedom.

Diplotype or haplotype effects on the trait, i.e., the existence of a QTN, can be tested using the following hypotheses expressed as
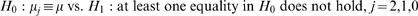
(17)The log-likelihood ratio test statistic (*LR*_2_) under these two hypotheses can be similarly calculated,
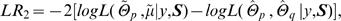
(18)where the tildes and hats denote the MLEs of parameters under the null and alternative hypotheses of (17), respectively. Although the critical threshold for determining the existence of a QTN can be based on empirical permutation tests, the *LR*_2_ may asymptotically follow a χ^2^ distribution with two degrees of freedom, so that the threshold can be obtained from the χ^2^ distribution table.

### Haplotyping a trait with multiple SNPs

**Haplotype structure:** The statistical method for QTN mapping is exemplified by a set of three SNPs, **S**_1_–**S**_3_, for a QTN. Two alleles 1 and 0 at each SNP are symbolized by *r*_1_, *r*_2_ and *r*_3_, respectively. Eight haplotypes, [111], [110], [101], [100], [011], [010], [001] and [000], formed by these three SNPs, have the frequencies arrayed in Θ*_p_* = (*p*_111,_ *p*_110,_ *p*_101,_ *p*_100,_ *p*_011,_ *p*_010,_ *p*_001,_ *p*_000_). Some genotypes are consistent with diplotypes, whereas the others that are heterozygous at two or more SNPs are not. Each double heterozygote contains two different diplotypes. One triple heterozygote, i.e., 10/10/10, contains four different diplotypes, [111][000] (in a probability of 2*p*_111_ *p*_000_), [110][001] (in a probability of 2*p*_110_ *p*_001_), [101][010] (in a probability of 2*p*_101_ *p*_010_) and [100][011] (in a probability of 2*p*_100_ *p*_011_). The relative frequencies of different diplotypes for this double or triple heterozygote are a function of haplotype frequencies (Table 2).

**Table 2:** Possible diplotypes and their frequencies for each of 27 genotypes at three SNPs within a QTN, and genotypic value vectors of composite diplotypes (assuming that [111] (*A*) is the risk haplotype and the others (*A̅*) are the non-risk haplotype).

Genotype	Diplotype			Composite diplotype	
	Configuration	Frequency	Relative frequency	Symbol	Mean
11/11/11	[111][111]	*P* ^2^_111_	1	*AA*	*μ*_2_
11/11/10	[111][110]	2*p*_111_ *p*_110_	1	*AA̅*	*μ*_1_
11/11/00	[110][110]	*P* ^2^_110_	1	*A̅* *A̅*	*μ*_0_
11/10/11	[111][101]	2*p*_111_ *p*_101_	1	*AA̅*	*μ*_1_
11/10/10	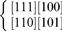	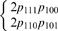	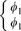	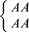	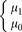
11/10/00	[110][100]	2*p*_110_ *p*_100_	1	*A̅* *A̅*	*μ*_0_
11/00/11	[101][101]	*P* ^2^_101_	1	*A̅* *A̅*	*μ*_0_
11/00/10	[101][100]	2*p* ^2^_101_ *p*_100_	1	*A̅* *A̅*	*μ*_0_
11/00/00	[100][100]	*P* ^2^_100_	1	*A̅* *A̅*	*μ*_0_
10/11/11	[111][011]	2*p*_111_ *p*_011_	1	*AA̅*	*μ*_1_
10/11/10	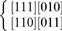	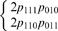	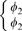	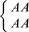	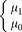
10/11/00	[110][010]	2*p*_110_ *p*_010_	1	*A̅* *A̅*	*μ*_0_
10/10/11	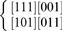	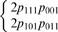	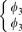	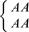	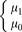
10/10/10	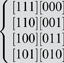	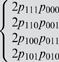	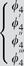	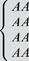	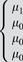
10/10/00	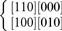	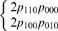	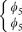	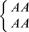	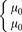
10/00/11	[101][001]	2*p*_101_ *p*_001_	1	*A̅* *A̅*	*μ*_0_
10/00/10	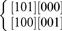	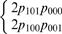	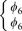	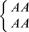	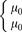
10/00/00	[100][000]	2*p*_100_ *p*_000_	1	*A̅* *A̅*	*μ*_0_
00/11/11	[011][011]	*P* ^2^_011_	1	*A̅* *A̅*	*μ*_0_
00/11/10	[011][010]	2*p*_011_ *p*_010_	1	*A̅* *A̅*	*μ*_0_
00/11/00	[010][010]	*P* ^2^_010_	1	*A̅* *A̅*	*μ*_0_
00/10/11	[011][001]	2*p*_011_ *p*_001_	1	*A̅* *A̅*	*μ*_0_
00/10/10	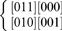	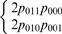	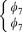	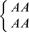	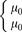
00/10/00	[010][000]	2*p*_010_ *p*_000_	1	*A̅* *A̅*	*μ*_0_
00/00/11	[001][001]	*P* ^2^_001_	1	*A̅* *A̅*	*μ*_0_
00/00/10	[001][000]	2*p*_001_ *p*_000_	1	*A̅* *A̅*	*μ*_0_
00/00/00	[000][000]	*P* ^2^_000_	1	*A̅* *A̅*	*μ*_0_

In the F_2_ population, there are 27 genotypes for the three SNPs. Let 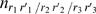 (*r*_1_≥*r*′_1_,*r*_2_≥*r*′_2_,*r*_3_≥*r*′_3_ = 1,0) be the number of offspring for a genotype. As seen in Table 2, the frequency of each genotype is expressed in terms of haplotype frequencies. Similar to equation (25), the phenotypic value of the trait for individual *i* is expressed, at the diplotype level, as
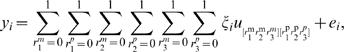
(19)where *ξ_i_* is the indicator variable defined as 1 if a diplotype considered is compatible with subject *i* and as 0 otherwise, 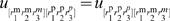 is the genotypic value for diplotype [*r*_1_^m^ *r*_2_^m^ *r*_3_^m^][*r*_1_^p^ *r*_2_^p^ *r*_3_^p^], and *e_i_* is the residual error distributed as *N*(0,*σ* ^2^). Note that **m** and **p** stand for the maternally and paternally derived alleles, respectively.

By assuming [111] as a risk haplotype (labelled by *A*) and all the others as non-risk haplotypes (labelled by *A̅*), Table 2 provides the formulation of genotypic values for three composite diplotypes, *μ*_2_ for *AA*, *μ*_1_ for *AA̅* and *μ*_0_ for *A̅* *A̅*. The haplotype effect parameters and residual covariance matrix are arrayed by a quantitative genetic parameter vector Θ*_q_* = (*μ*_2_, *μ*_1_, *μ*_0_,*σ* ^2^).

**Likelihoods and algorithms:**With the above notation, we construct two likelihoods, one for haplotype frequencies (Θ*_p_*) based on SNP data (**S**) and the other for quantitative genetic parameters (Θ*_q_*) based on haplotype frequencies (Θ*_p_*), phenotypic (*y*) and SNP data (**S**). They are, respectively, expressed as
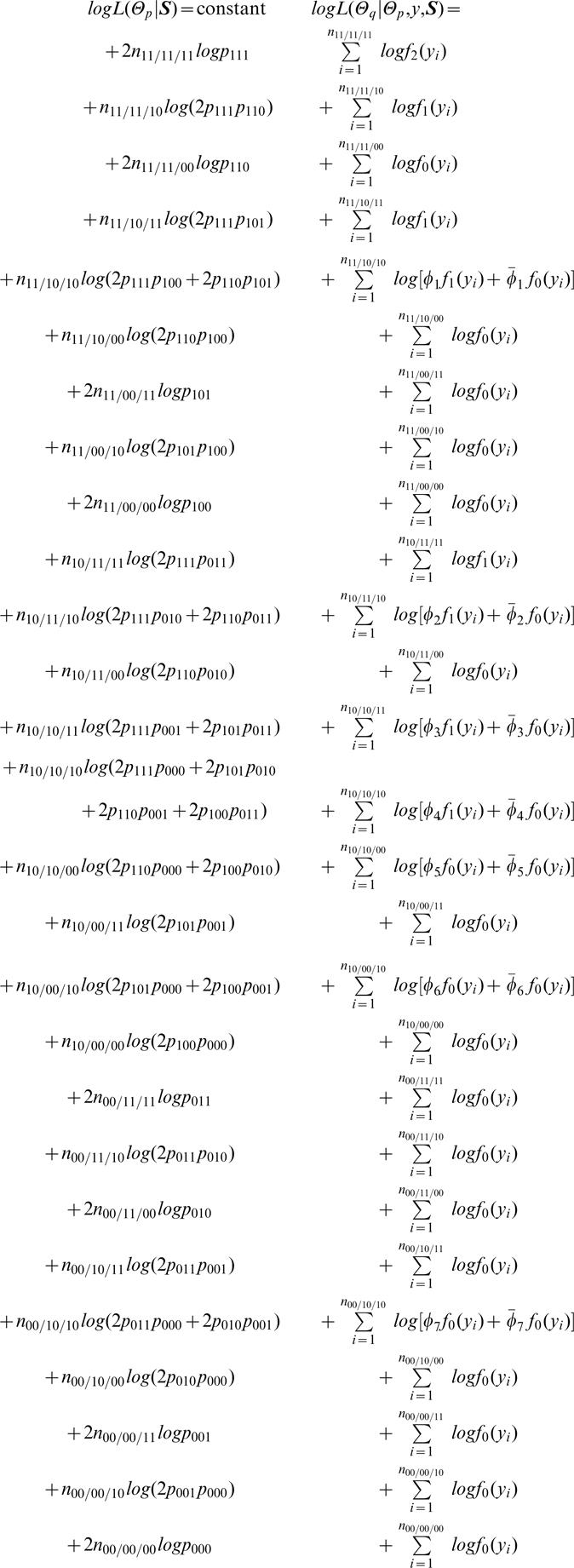
(20)where *φ*_._'s (*φ̅*. = 1−*φ*) are defined below, and *f _j _( y_j_)* (*j* = 2, 1, 0) is a normal distribution density function of composite diplotype *j*.

A two-stage hierarchical EM algorithm is derived to estimate haplotype frequencies and quantitative genetic parameters. At the higher hierarchy of the EM framework, we calculate the proportions of a particular diplotype within double or triple heterozygous genotypes (E step) by
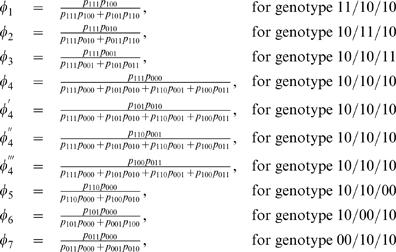
(21)The calculated relative proportions by equation (21) were used to estimate the haplotype frequencies with
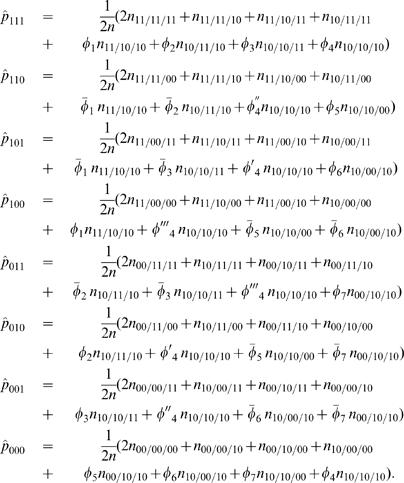
(22)


At the lower hierarchy of the EM framework, we calculate the posterior probabilities of a double or triple heterozygous individual *i* to be a particular diplotype (*A̅*) (E step), for which where [111] is assumed as the risk haplotype, expressed as
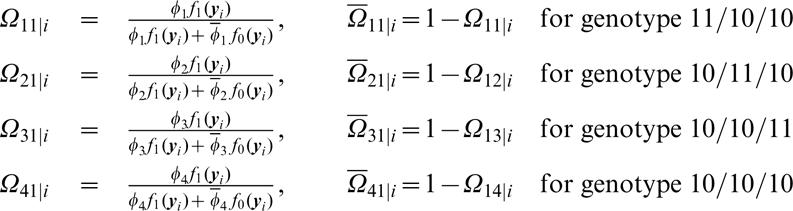
(23)With the calculated posterior probabilities by the above equation (23), we then estimate the quantitative genetic parameters, Θ*_q_*, based on the log-likelihood equations. These equations have similar, but more complicated, forms like equations (12) and (13).

Hypothesis tests can be made for linkage disequilibria among three SNPs and haplotype effects. Four different linkage disequilibria, *D*_12_, *D*_13_, *D*_23_ and *D*_123_, that describe the linkage among three SNPs can each be tested using the null hypotheses described by equation (21). The log-likelihood ratios for each hypothesis are thought to follow a χ^2^ distribution.

***R*** **-SNP model:** The idea for haplotyping a quantitative trait is described for two- and three-SNP models. It is possible that these models are too simple to characterize genetic variants for quantitative variation. With the analytical line for the two- and three-SNP sequencing model, a model can be developed to include an arbitrary number of SNPs whose sequences are associated with the phenotypic variation. A key issue for the multi-SNP sequencing model is how to distinguish among 2*^r^* ^−1^ different diplotypes for the same genotype heterozygous at *r* loci. The relative frequencies of these diplotypes can be expressed in terms of haplotype frequencies. The integrative EM algorithm can be employed to estimate the MLEs of haplotype frequencies. A general formula for estimating haplotype frequencies can be derived.

## Results

The statistical model described above can be used to map and identify QTNs for a quantitative trait in an F_2_ population. Because the marker data we have for mouse are microsatellites rather than SNPs, we use these microsatellite markers as a surrogate of SNPs for the purpose to demonstrate the utility of the model. Our marker data were from Vaughn et al.'s [21] study in which a linkage map composed of 19 chromosomes was constructed with 96 microsatellite markers for 502 F_2_ mice (259 males and 243 females) derived from two strains, the Large (LG/J) and Small (SM/J). This map has a total map distance of ∼1780 cM (in Haldane's units) and an average interval length of ∼23 cM. The F_2_ progeny was measured for their body mass at 10 weekly intervals starting at age 7 days. The raw weights were corrected for the effects of each covariate due to dam, litter size at birth, parity and sex [21]. Here, only adult body weights at week 10 are used for “QTN” analysis.

For each F_2_ mouse, the parental origin of alleles at each marker can be discerned in molecular studies. Let *L* and *S* be the alleles inherited from the Large (LG/J) and Small (SM/J) strains, respectively. For any pair of markers, there are four different haplotypes, *LL*, *LS*, *SL* and *SS*, whose frequencies are accordingly denoted as
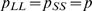
and 
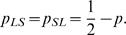
By assuming all the four haplotypes as a risk haplotype, respectively, the above model allows for the estimates of haplotype frequencies by the EM iteration at the higher hierarchy and of composite genotypic values by the EM iteration at the lower hierarchy. The estimated haplotype frequencies are used to estimate linkage disequilibrium based on equation (14) and the recombination fraction (*r*) based on equation (4). This estimation process is moved from the first (**M**_1_–**M**_2_) to last pair of markers (**M**_6_–**M**_7_) on chromosome 1 and then from chromosome 1 to 19.

Table 3 tabulates the results of the MLEs of haplotype frequencies and log-likelihoods under the assumptions of different risk haplotypes. A total of 96 markers are sparsely located on 19 mouse chromosomes, with the estimated recombination fractions from the linkage disequilibrium model [8] consistent with those obtained from the linkage model [21]. Significant likelihood ratios for testing haplotype effects were determined by critical values obtained from the *χ* ^2^-square distribution with two degrees of freedom with a Bonferroni adjustment to the type I error. The adjusted critical values for the two- and three-marker QTN models are 18.20 and 18.76, respectively, at the 5% significance level. Significant haplotype effects are detected for a total of eight marker pairs (Table 3), which include one pair on chromosome 4, two consecutive pairs on chromosome 6, four consecutive pairs on chromosome 7 and one pair on chromosome 14. For some pairs, multiple significant risk haplotypes were detected. Risk haplotypes purely composed of alleles inherited from the LG/J or SM/J parent exert a positive or negative additive effect on body weight, respectively. Based on the relative values of estimated additive and dominant effects, the significant marker pairs detected display partial dominant effects (Table 3).

**Table 3:** The MLEs of haplotype frequencies and significant log-likelihood ratios (LR) by assuming different risk haplotypes in the F_2_ population of mice.

Marker pair	Association		Selection of risk haplotype			Haplotype effect	
	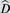	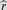	Risk haplotype	Frequency	LR_2_	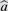	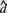
**D**4**Mit**16-**D**4m**Mit**13	0.16	0.17	*LL*	0.36	157.59	0.53	0.46
			*LS*	0.08	152.57	0.60	−0.91
			*SL*	0.10	155.55	−1.46	0.90
			*SS*	0.47	153.26	−0.35	0.18
**D**6**Mit**9-**D**6**Nds**5	0.18	0.14	*LL*	0.42	19.95	1.17	0.24
**D6Nds**5–**D**6**Mit**15	0.14	0.22	*LL*	0.38	25.14	1.25	0.44
			*SS*	0.41	37.98	−1.69	0.51
**D**7**Mit2**1–**D**7**Nds**1	0.09	0.32	*LL*	0.34	30.84	0.93	1.41
			*SS*	0.34	36.54	−1.70	−0.07
**D**7**Nds**1–**D**7**Mit17**	0.19	0.12	*LL*	0.44	46.87	1.66	0.59
			*SS*	0.45	43.31	−1.75	0.50
**D**7**Mit17**–**D**7**Mit9**	0.19	0.12	*LL*	0.43	33.41	1.42	0.57
			*SS*	0.45	34.35	−1.47	0.99
**D**7**Mit9**–**D**7**Nds**4	0.12	0.26	*SS*	0.38	19.84	−1.15	1.09
**D**14**Mit5**–**D**14**Mit7**	0.17	0.16	*LL*	0.43	19.35	1.10	0.33

The results were obtained by using a two-SNP QTN model.

The results from the three-marker model are basically consistent with those from the two-marker model (Table 4). The advantage of the three-marker model is that it incorporates the interferences between adjacent marker intervals into the estimation process and, thus, can potentially increase the estimation precision of haplotype effects.

**Table 4:** The MLEs of haplotype frequencies and significant log-likelihood ratios (LR) by assuming different risk haplotypes in the F_2_ population of mice.

Marker pair	Selection of risk haplotype			Haplotype effect	
	Risk haplotype	Frequency	LR_2_	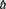	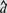
**D**4**Mit**45–**D**4**Mit**16–**D**4**Mit**13	*LLL*	0.29	124.70	0.40	0.61
	*LLS*	0.07	121.34	1.08	−1.00
	*LSL*	0.01	122.58	−1.88	−2.56
	*LSS*	0.07	122.08	−0.44	1.40
	*SLL*	0.07	122.14	0.80	0.12
	*SLS*	0.01	132.86	-	-
	*SSL*	0.09	122.33	−1.32	1.09
	*SSS*	0.40	123.65	−0.55	0.28
**D**6**Mit**9–**D**6**Nds**5–**D**6**Mit**15	*SSS*	0.34	22.65	−1.51	0.39
**D**7**Mit**21–**D**7**Nds**1–**D**7**Mit**17	*LLL*	0.29	38.28	0.81	1.85
	*SSS*	0.30	33.74	−1.80	0.09
**D**7**Nds**1–**D**7**Mit**17–**D**7**Mit**9	*LLL*	0.38	34.39	1.48	0.47
	*SSS*	0.40	32.18	−1.61	0.61
**D**7**Mit**17–**D**7**Mit**9–**D**7**Nds**4	*LLL*	0.33	21.74	1.20	0.45
	*SSS*	0.33	29.41	−1.60	1.36
**D**14**Nds**1–**D**14**Mit**5–**D**14**Mit**7	*LLL*	0.30	19.55	1.44	−0.50

The results were obtained by using a three-SNP QTN model.

## Discussion

Quantitative trait locus (QTL) mapping aims to identify narrow chromosomal segments for a quantitative trait by using a statistical method, and has proven its value to study the genetic architecture of the trait in a variety of species [6]–[8]. The limitations of this technique lie in its inability to characterize the structure and organization of DNA sequences and statistical difficulty in deriving the distribution of test statistics under the null hypothesis of no QTL [22]. At least partly for these reasons, despite thousands of QTL reported for different traits and populations, a very small portion of them have been cloned [9]. With the completion of the genome projects for several important organisms, a new line of thought in the post genomic era has begun to emerge for the identification of specific combinations of nucleotides or haplotypes that contribute to a complex quantitative trait [13], [23].

Theory and methods for haplotype discovery have well been established for natural populations [13] in which the non-random association among different single nucleotide polymorphims (SNP), specified by the coefficients of linkage disequilibria, lays a foundation for the mixture model of haplotyping a quantitative trait. In this article, we derived a statistical model for detecting haplotypes and estimating their effects on quantitative variation of a trait in experimental crosses. We used the principle of linkage disequilibrium analysis to characterize the linkage among different markers that is usually described by the recombination fractions in a commonly used F_2_ population, initiated with two inbred lines. We established an interchangeable relationship between the linkage and linkage disequilibrium. The merit of this relationship in trait haplotyping includes the incorporation of interferences between adjacent marker intervals into the estimation and test of haplotype effects when multiple markers are modelled simultaneously.

The haplotyping model developed in this article was used to analyze a published F_2_ population of mouse [21], but we used microsatellite markers as the surrogate of SNPs so that we can detect the effects of haplotypes constructed by microsatellite alleles. The whole-genome of mouse was scanned for haplotype effects on body weight by a two- and multi-marker model, respectively. Consistent results were observed from the two models, which suggests that four regions in mouse chromosomes 4, 6, 7, and 14 contribute to variation in body weight. These findings are in a good agreement with those from traditional interval QTL mapping [21]. But our haplotype discovery is more informative in terms of the characterization of specific haplotype structure and organization responsible for trait variation.

We have proposed a new model for haplotyping a quantitative trait in the F_2_ progeny population. The tenet of the model can be extended to haplotype a complicated trans-generational pedigree, founded with multiple original parents and involving individuals with different relatedness. The model can also be modified to dissect the epistatic effects of different genes [23] and the interaction of genes with environment. For these extensions, haplotype selection aimed to detect the risk haplotypes that are expressed differently from the others present many challenges, but is crucial for the facilitation of the process of detecting the association between haplotype diversity and phenotypic variation.

Our haplotyping model offers a powerful tool for positional cloning of QTL that are important for a complex trait. Flint et al. [9] reviewed the potential of currently available cloning strategies, such as probabilistic ancestral haplotype reconstruction, Yin-Yang crosses and in silico analysis of sequence variants, to identify genes that underlie QTL in rodents. Our model, in conjunction with these strategies, may open a new gateway for the illustration of a detailed picture of the genetic architecture for a complex trait.
